# Choice of the initial antiretroviral treatment for HIV-positive individuals in the era of integrase inhibitors

**DOI:** 10.1371/journal.pone.0221598

**Published:** 2019-08-26

**Authors:** Belén Alejos, Inés Suárez-García, Otilia Bisbal, José Antonio Iribarren, Víctor Asensi, Miguel Górgolas, Roberto Muga, Santiago Moreno, Inma Jarrín, CoRIS cohort

**Affiliations:** 1 Centro Nacional de Epidemiología, Instituto de Salud Carlos III, Madrid, Spain; 2 Infectious Diseases Unit, Department of Internal Medicine, Hospital Universitario Infanta Sofia, Madrid, Spain; 3 Universidad Europea, Madrid, Spain; 4 Hospital Universitario Doce de Octubre, Madrid, Spain; 5 Hospital Universitario de Donostia, Donostia, Spain; 6 Hospital Universitario Central de Asturias, Oviedo, Spain; 7 Hospital Universitario Fundación Jiménez Díaz, Madrid, Spain; 8 Hospital Universitari Germans Trias i Pujol, Badalona, Spain; 9 Hospital Universitario Ramón y Cajal-IRYCIS, Madrid, Spain; Institut Hospital del Mar d'Investigacions Mediques, SPAIN

## Abstract

**Background:**

We aimed to describe the most frequently prescribed initial antiretroviral therapy (ART) regimens in recent years in HIV-positive persons in the Cohort of the Spanish HIV/AIDS Research Network (CoRIS) and to investigate factors associated with the choice of each regimen.

**Methods:**

We analyzed initial ART regimens prescribed in adults participating in CoRIS from 2014 to 2017. Only regimens prescribed in >5% of patients were considered. We used multivariable multinomial regression to estimate Relative Risk Ratios (RRRs) for the association between sociodemographic and clinical characteristics and the choice of the initial regimen.

**Results:**

Among 2874 participants, abacavir(ABC)/lamivudine(3TC)/dolutegavir(DTG) was the most frequently prescribed regimen (32.1%), followed by tenofovir disoproxil fumarate (TDF)/emtricitabine (FTC)/elvitegravir(EVG)/cobicistat(COBI) (14.9%), TDF/FTC/rilpivirine (RPV) (14.0%), tenofovir alafenamide (TAF)/FTC/EVG/COBI (13.7%), TDF/FTC+DTG (10.0%), TDF/FTC+darunavir/ritonavir or darunavir/cobicistat (bDRV) (9.8%) and TDF/FTC+raltegravir (RAL) (5.6%).

Compared with ABC/3TC/DTG, starting TDF/FTC/RPV was less likely in patients with CD4<200 cells/μL and HIV-RNA>100.000 copies/mL. TDF/FTC+DTG was more frequent in those with CD4<200 cells/μL and HIV-RNA>100.000 copies/mL. TDF/FTC+RAL and TDF/FTC+bDRV were also more frequent among patients with CD4<200 cells//μL and with transmission categories other than men who have sex with men. Compared with ABC/3TC/DTG, the prescription of other initial ART regimens decreased from 2014–2015 to 2016–2017 with the exception of TDF/FTC+DTG. Differences in the choice of the initial ART regimen were observed by hospitals’ location.

**Conclusions:**

The choice of initial ART regimens is consistent with Spanish guidelines’ recommendations, but is also clearly influenced by physician’s perception based on patient’s clinical and sociodemographic variables and by the prescribing hospital location.

## Introduction

International and local guidelines for the treatment of HIV-infection provide recommendations on the preferred drug combinations for initial antiretroviral therapy (ART) of treatment-naïve patients [1,2]. Although there is a wide range of highly effective and well tolerated therapies, most recent guidelines in Spain and the United States have limited preferred options to integrase inhibitor-based regimens based on the results of clinical trials as well as on the advantages of individual drugs [3,4]. Other guidelines such as the ones from the European AIDS Clinical Society also include regimens based on rilpivirine and boosted darunavir as preferred [2]. Some experts and clinicians feel that current recommendations might be too restrictive and regimens other than those based on integrase inhibitors would be at least as good choices to initiate therapy in most patients.

Previous studies have shown that the decision on what specific ART regimen is prescribed to each patient can be influenced by a variety of factors, not only dependent on the patient (such as comorbidities, HIV stage, concerns about toxicity or drug interactions, risk of nonadherence, patient’s preference) but also on the prescribing physician (such as HIV treatment experience, budget limitations, hospital’s characteristics, physician’s preference) [5,6]. The few studies that have investigated the factors influencing the choice of initial ART were published before newer drugs such as rilpivirine and integrase inhibitors were widely used [5,7–9], and there is no evidence on the factors that could influence the choice of ART with the more recent treatment regimens, and specifically with those including an integrase inhibitor.

In this study, we aimed to describe the most frequently prescribed initial ART regimens in recent years in HIV-positive patients in the Cohort of the Spanish HIV/AIDS Research Network (CoRIS) and to investigate factors associated with the choice of initial ART.

## Methods

### Study design

CoRIS is an open, multicentre, prospective cohort of ART-naïve HIV-positive adults recruited in 45 centres from 13 of the 17 Autonomous Regions of Spain. Patients are followed periodically in accordance with routine clinical practice. Data are subject to internal quality control. A complete description of the cohort has been published elsewhere [10,11].

### Study population

Patients included were antiretroviral-naïve, aged ≥ 18 years, and had started ART between 1^st^ September 2014 (when DTG became available in Spain) and 30^th^ November 2017 with the most commonly used first-line antiretroviral regimens. In order to facilitate the analyses, only regimens prescribed in >5% of individuals were considered. Patients with no follow-up after initiation of ART were excluded.

### Definition of variables

The primary endpoint was the choice of the initial ART according to the most frequently prescribed regimens: abacavir (ABC) /lamivudine (3TC) /dolutegavir (DTG) was the most frequently prescribed ART, followed by tenofovir disoproxil fumarate (TDF) /emtricitabine (FTC)/ elvitegravir (EVG)/ cobicistat (COBI), TDF/FTC/rilpivirine (RPV), tenofovir alafenamide(TAF)/FTC/EVG/COBI, TDF/FTC+DTG, TDF/FTC+darunavir/ritonavir or darunavir/cobicistat (bDRV) and TDF/FTC+raltegravir (RAL).

Possible explanatory variables included sex (male, female), age at ART initiation (<30, 30–49, ≥50 years), transmission category (men who have sex with men [MSM], heterosexual, injecting drug use, other, unknown), educational level (no education or compulsory education, upper secondary or university education, unknown), origin (Spain, immigrants, unknown), CD4 T-cell count (<200, ≥200 cells/μL, unknown) and viral load (≤100,000, >100,000 copies/mL, unknown), presence of hepatitis C virus antibodies (no, yes, unknown), presence of hepatitis B virus surface antigen (HBVSA) (no, yes, unknown), triglycerides (≤150, >150 mg/dL, unknown), HDL (≤40, >40 mg/dL, unknown) and total cholesterol (≤200, >200 mg/dL, unknown) within 6 months previous to ART initiation, AIDS diagnosis at ART initiation (no, yes), presence of comorbidities (cardiovascular, nephrology, neurologic [no, yes]), period of ART initiation (2014–2015,2016–2017) and hospital characteristics (number of beds [<500, ≥500] and hospital’s location [Autonomous region]).

### Statistical analysis

Descriptive analysis of patients’ characteristics was carried out using frequency tables for categorical variables and median and interquartile range for continuous variables. Differences in socio-demographic and clinical characteristics according to initial regimen were assessed with the non-parametric Kruskal-Wallis test for continuous variables and the chi-squared test for independence for categorical variables. We used multivariable multinomial regression to estimate Relative Risk Ratios (RRRs) for the association between explanatory variables and the choice of the initial regimen. All variables that retained a significant independent association (p <0.05) were included in the final model.

Heterogeneity introduced by different hospitals was accounted for by including the study hospital as a fixed effect in the model and by using robust methods to estimate standard errors and, thus, to calculate 95% confidence intervals and p-values.

All statistical analyses were performed using Stata software (version 15.0; Stata Corporation, College Station, Texas, USA).

### Ethics

Ethics approval was obtained from all hospitals’ Ethics’ Committees and every patient provided written informed consent to participate in the cohort. This study was approved by the Ethics Committee of Instituto de Salud Carlos III (CEI PI 63_2017-v2).

## Results

Between September 1, 2014 and November 30, 2017, 3,575 patients aged ≥18 years initiated treatment; among these, 147 (4.1%) with no follow-up after ART initiation and 554 (15.5%) who initiated a treatment prescribed in <5% of patients were excluded. The final analysis included 2,874 (80.4%) subjects who initiated ART during the period of study. They were predominantly male (88.5%) and 59.7% were from Spain. Transmission route was heterosexual contact in 23.5% and MSM in 68.9%. At treatment initiation, median age was 36 years (interquartile range [IQR]: 30–44), median CD4+ T-cell count was 416 cells/μL (IQR: 243–591), 8.5% of patients had a history of AIDS diagnosis, 34.5% had a viral load >100,000 copies/mL and 89.2% were attending hospitals with more than 500 beds.

The most frequently prescribed initial regimens in the study period and the differences in sociodemographic and clinical characteristics of patients according to their initial ART regimen are shown in Table 1. TDF/FTC+bDRV and TDF/FTC+RAL were significantly less often used in participants with upper education and among MSM. TAF/FTC/EVG/COBI was more likely prescribed among immigrants and TDF/FTC+RAL among those aged 50 years or older.

**Table 1:** Baseline characteristics of 2,874 study participants according to the initial antiretroviral regimen.

	ABC/3TC/DTG	TDF/FTC/EVG/COBI	TDF/FTC/RPV	TAF/FTC/EVG/COBI	TDF/FTC+DTG	TDF/FTC+bDRV	TDF/FTC+RAL	P
	923 (32.1%)	427 (14.9%)	401 (14.0%)	394 (13.7%)	287 (10.0%)	282 (9.8%)	160 (5.6%)	
**Male sex**	815 (88.3%)	393 (92.0%)	365 (91.0%)	345 (87.6%)	253 (88.2%)	248 (87.9%)	125 (78.1%)	<0.001
**Upper education**	518 (56.1%)	242 (56.7%)	215 (53.6%)	232 (58.9%)	155 (54.0%)	125 (44.3%)	70 (43.8%)	0.033
**Immigrants**	339 (36.7%)	172 (40.3%)	151 (37.7%)	191 (48.5%)	109 (38.0%)	101 (35.8%)	61 (38.1%)	0.003
**Men who have sex with men**	659 (71.4%)	324 (75.9%)	289 (72.1%)	278 (70.6%)	191 (66.6%)	154 (54.6%)	84 (52.5%)	<0.001
**Age ≥50 years-old**	117 (12.7%)	33 (7.7%)	40 (10.0%)	41 (10.4%)	45 (15.7%)	45 (16.0%)	31 (19.4%)	<0.001
**AIDS**	53 (5.7%)	28 (6.6%)	3 (0.7%)	19 (4.8%)	54 (18.8%)	41 (14.5%)	47 (29.4%)	<0.001
**Median CD4 (IQR)**	443 (283–609)	445 (274–590)	506 (378–719)	430 (265–591)	286 (94–473)	296 (111–492)	230 (72–449)	<0.001
**CD4 count <200 cells/μL**	120 (13.0%)	71 (16.6%)	16 (4.0%)	65 (16.5%)	107 (37.3%)	94 (33.3%)	68 (42.5%)	<0.001
**Viral load >100,000 copies/mL**	317 (34.3%)	147 (34.4%)	10 (2.5%)	154 (39.1%)	155 (54.0%)	131 (46.5%)	77 (48.1%)	<0.001
**Triglycerides >150 mg/dL**	154 (16.7%)	65 (15.2%)	54 (13.5%)	62 (15.7%)	69 (24.0%)	48 (17.0%)	39 (24.4%)	<0.001
**HDL cholesterol ≤40 mg/dL**	419 (45.4%)	147 (34.4%)	128 (31.9%)	168 (42.6%)	139 (48.4%)	129 (45.7%)	74 (46.3%)	<0.001
**Total cholesterol >200 mg/dL**	113 (12.2%)	49 (11.5%)	47 (11.7%)	49 (12.4%)	27 (9.4%)	32 (11.3%)	21 (13.1%)	0.941
**HCV + antibody**	46 (5.0%)	21 (4.9%)	23 (5.7%)	19 (4.8%)	16 (5.6%)	19 (6.7%)	14 (8.8%)	0.073
**HBV S + antigen**	3 (0.3%)	11 (2.6%)	13 (3.2%)	6 (1.5%)	12 (4.2%)	5 (1.8%)	5 (3.1%)	0.551
**Cardiovascular event**	14 (1.5%)	9 (2.1%)	5 (1.2%)	6 (1.5%)	9 (3.1%)	5 (1.8%)	11 (6.9%)	<0.001
**Renal event**	17 (1.8%)	10 (2.3%)	5 (1.2%)	8 (2.0%)	7 (2.4%)	3 (1.1%)	7 (4.4%)	0.258
**Neurologic event**	33 (3.6%)	14 (3.3%)	12 (3.0%)	8 (2.0%)	15 (5.2%)	8 (2.8%)	10 (6.3%)	0.151
**Hospital size < = 500 beds**	834 (90.4%)	377 (88.3%)	352 (87.8%)	340 (86.3%)	268 (93.4%)	250 (88.7%)	143 (89.4%)	0.081
Hospitals’ location*								<0.001
A	123 (13.3%)	118 (27.6%)	67 (16.7%)	124 (31.5%)	22 (7.7%)	26 (9.2%)	17 (10.6%)	
B	2 (0.2%)	1 (0.2%)	3 (0.7%)	2 (0.5%)	0 (0.0%)	2 (0.7%)	0 (0.0%)	
C	11 (1.2%)	0 (0.0%)	5 (1.2%)	1 (0.3%)	6 (2.1%)	17 (6.0%)	0 (0.0%)	
D	9 (1.0%)	1 (0.2%)	2 (0.5%)	0 (0.0%)	4 (1.4%)	4 (1.4%)	0 (0.0%)	
E	87 (9.4%)	62 (14.5%)	36 (9.0%)	36 (9.1%)	16 (5.6%)	27 (9.6%)	18 (11.3%)	
F	22 (2.4%)	12 (2.8%)	19 (4.7%)	5 (1.3%)	5 (1.7%)	2 (0.7%)	6 (3.8%)	
G	104 (11.3%)	19 (4.4%)	21 (5.2%)	26 (6.6%)	9 (3.1%)	9 (3.2%)	0 (0.0%)	
H	22 (2.4%)	0 (0.0%)	7 (1.7%)	0 (0.0%)	20 (7.0%)	3 (1.1%)	1 (0.6%)	
I	407 (44.1%)	159 (37.2%)	131 (32.7%)	146 (37.1%)	138 (48.1%)	56 (19.9%)	49 (30.6%)	
J	40 (4.3%)	19 (4.4%)	6 (1.5%)	17 (4.3%)	26 (9.1%)	15 (5.3%)	6 (3.8%)	
K	25 (2.7%)	0 (0.0%)	12 (3.0%)	3 (0.8%)	0 (0.0%)	23 (8.2%)	21 (13.1%)	
L	5 (0.5%)	0 (0.0%)	56 (14.0%)	0 (0.0%)	0 (0.0%)	67 (23.8%)	32 (20.0%)	
M	66 (7.2%)	36 (8.4%)	36 (9.0%)	34 (8.6%)	41 (14.3%)	31 (11.0%)	10 (6.3%)	
**Period of ART initiation- 2016–17**	657 (71.2%)	116 (27.2%)	89 (22.2%)	391 (99.2%)	194 (67.6%)	128 (45.4%)	67 (41.9%)	<0.001

*Each capital letter identifies one of the Autonomous Regions. The names of the Autonomous Regions cannot be disclosed due to confidentiality reasons

As expected, rilpivirine was rarely used in patients with high baseline viral load >100,000 copies/mL (2.5%) or CD4 counts <200 cells/μL (4.0%). A higher proportion of the patients starting TDF/FTC+DTG, TDF/FTC+bDRV and TDF/FTC+RAL had a CD4 cell count below 200 cells/μL and an AIDS diagnosis at start of treatment. TDF/FTC+DTG and TDF/FTC+RAL were more frequent in participants with triglycerides >150 mg/dL and cholesterol-HDL ≤40 mg/dL. Large differences in the prescription of initial regimen were noted by hospital location but not by hospital size. There were also differences in the use of initial regimen in relation to HBVSA and previous cardiovascular event; nevertheless they must be interpreted with caution as the number of events was small.

**Fig 1:**
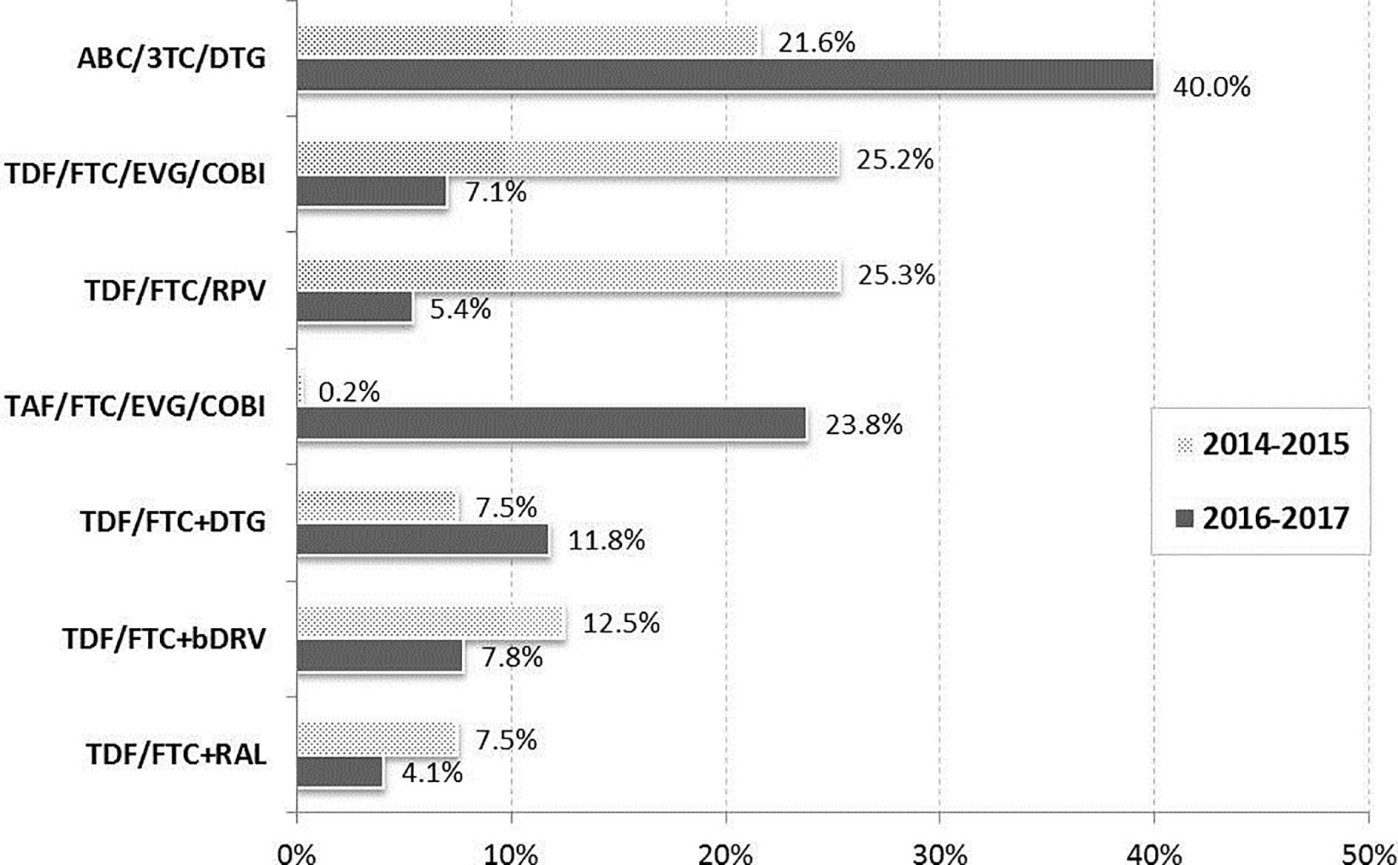
Distribution of the initial antiretroviral treatment by period of ART initiation.

We also observed changes over time in the distribution of the most frequent initial ART regimens (Fig 1). In the period 2014–2015, TDF/FTC/RPV (25.3%) and TDF/FTC/EVG/COBI (25.2%) were the most frequently prescribed initial ART, followed by ABC/3TC/DTG (21.6%). However, in the period 2016–2017, the most frequent regimen was ABC/3TC/DTG (40.0%), followed by TAF/FTC/EVG/COBI (23.8%). When grouping the regimens by their third agent, the only regimens whose prescription increased over time were the ones based in DTG (including ABC/3TC/DTG and TDF/FTC+DTG) and EVG (including TDF/FTC/EVG/COBI and TAF/FTC/EVG/COBI), while the prescription of all other regimens decreased. Regimens based in DTG increased from being prescribed in 29.1% of the patients in 2014–2015 to 51.8% in 2016–2017, and those based in EVG increased from 25.2% to 30.9%, respectively (Fig 1).

Results from multivariable analysis are shown in Table 2. Compared with ABC/3TC/DTG, starting TDF/FTC/RPV was less likely in patients with CD4 counts <200 cells/μL (RRR, 95% CI: 0.41, 0.25–0.67) and with HIV-RNA >100.000 copies/mL (RRR, 95% CI: 0.05, 0.03–0.10); inversely TDF/FTC+DTG was more frequent in those with CD4 counts <200 cells/μL (RRR, 95% CI: 3.35, 2.34–4.80) and with HIV-RNA >100.000 copies/mL (RRR, 95% CI: 1.74, 1.21–2.52). TDF/FTC+RAL and TDF/FTC+bDRV were also more likely to be prescribed among patients with CD4 counts <200 cells/μL (RRR, 95% CI: 3.76, 2.39–5.92 and RRR, 95% CI: 2.61, 1.51–4.49, respectively) and those with transmission categories other than MSM (RRR, 95% CI: 2.00, 1.29–3.10 and RRR, 95% CI: 1.73, 1.14–2.61, respectively). Compared with ABC/3TC/DTG, the prescription of other initial ART regimens decreased over time (p<0.001), with the exception of TDF/FTC+DTG (p = 0.173). Valid conclusions regarding changes over time in the prescription of TAF/FTC/EVG/COBI could not be drawn because it was widely available only after 2016. Overall differences in the choice of the initial ART regimen were observed by hospital’s location. No other significant factors were found for the choice of TDF/FTC/EVG/COBI and TAF/FTC/EVG/COBI, compared to ABC/3TC/DTG.

**Table 2:** Multivariable relative risk ratios for predictors of the choice of the initial ART regimen relative to ABC/3TC/DTG.

	TDF/FTC/EVG/COBI		TDF/FTC/RPV		TAF/FTC/EVG/COBI		TDF/FTC+DTG		TDF/FTC+bDRV		TDF/FTC+RAL	
	RRR (IC 95%)	P	RRR (IC 95%)	P	RRR (IC 95%)	P	RRR (IC 95%)	P	RRR (IC 95%)	P	RRR (IC 95%)	P
**HIV transmission category**												
MSM	1		1		1		1		1		1	
Non-MSM	0.96 (0.67–1.39)	0.833	1.10 (0.79–1.54)	0.575	1.10 (0.78–1.54)	0.598	0.98 (0.69–1.38)	0.892	1.73 (1.14–2.61)	0.009	2.00 (1.29–3.10)	0.002
**CD4 T-cell count**												
> = 200 cells/μL	1		1		1		1		1		1	
<200 cells/μL	1.28 (0.97–1.70)	0.085	0.41 (0.25–0.67)	<0.001	1.18 (0.84–1.66)	0.331	3.35 (2.34–4.80)	<0.001	2.61 (1.51–4.49)	0.001	3.76 (2.39–5.92)	<0.001
**Viral Load**												
< = 100,000 cop/mL	1		1		1		1		1		1	
>100,000 cop/mL	1.03 (0.72–1.48)	0.869	0.05 (0.03–0.10)	<0.001	1.15 (0.87–1.52)	0.330	1.74 (1.21–2.52)	0.003	1.21 (0.84–1.75)	0.300	1.16 (0.70–1.91)	0.566
**Period of ART initiation**												
2014–2015	1		1		1		1		1		1	
2016–2017	0.17 (0.12–0.22)	<0.001	0.09 (0.05–0.15)	<0.001	NA		0.79 (0.57–1.11)	0.173	0.22 (0.12–0.42)	<0.001	0.23 (0.12–0.42)	<0.001
Hospitals’ location*		<0.001		<0.001		<0.001		<0.001		<0.001		<0.001
A	1		1		1		1		1		1	
B	1.17 (0.84–1.63)		7.02 (4.87–10.12)		0.57 (0.39–0.85)		NA		9.25 (4.43–19.32)		NA	
C	NA		2.06 (0.71–5.95)		0.05 (0.02–0.12)		3.96 (1.30–12.03)		10.54 (3.34–33.30)		NA	
D	0.22 (0.15–0.31)		0.98 (0.73–1.31)		NA		3.38 (1.85–6.18)		4.34 (2.14–8.79)		NA	
E	1.08 (0.57–2.04)		1.37 (0.35–5.42)		0.28 (0.16–0.51)		1.00 (0.39–2.55)		1.60 (0.45–5.64)		1.53 (0.69–3.40)	
F	0.70 (0.21–2.34)		2.12 (1.31–3.43)		0.18 (0.07–0.44)		1.28 (0.66–2.51)		0.43 (0.03–6.69)		1.78 (0.89–3.56)	
G	0.28 (0.21–0.39)		0.71 (0.55–0.93)		0.18 (0.12–0.27)		0.54 (0.31–0.93)		0.50 (0.27–0.92)		NA	
H	NA		1.10 (0.83–1.46)		NA		5.87 (3.38–10.17)		0.75 (0.39–1.42)		0.38 (0.18–0.78)	
I	0.60 (0.30–1.20)		1.08 (0.73–1.61)		0.26 (0.13–0.51)		2.01 (0.93–4.36)		0.85 (0.39–1.87)		1.15 (0.47–2.84)	
J	0.80 (0.39–1.66)		0.63 (0.32–1.21)		0.30 (0.12–0.74)		3.59 (1.45–8.86)		2.15 (1.06–4.35)		1.38 (0.42–4.53)	
K	NA		2.23 (1.61–3.08)		0.08 (0.05–0.12)		NA		5.12 (2.67–9.82)		6.97 (3.32–14.65)	
L	NA		78.87 (28.19–220.65)		NA		NA		101.92 (17.62–589.58)		71.74 (26.53–194.01)	
M	0.77 (0.25–2.43)		1.60 (0.60–4.27)		0.39 (0.15–1.01)		3.73 (1.49–9.33)		2.66 (0.73–9.74)		1.25 (0.32–4.88)	

*Each capital letter identifies one of the Autonomous Regions. The names of the Autonomous Regions cannot be disclosed due to confidentiality reasons

## Discussion

This is, to our knowledge, the first study assessing the factors influencing the choice of initial ART in a reasonable large cohort in recent years, when newer treatment regimens have become widely available. All the regimens analyzed were considered preferred or alternative by the Spanish treatment guidelines during the study period [1,12–14], but our results suggest that the choice between different regimens could be influenced by certain characteristics of the patient or the hospitals where the treatment is initiated, in addition to the efficacy and toxicity results shown in clinical trials.

Overall, ABC/3TC/DTG was the most frequently prescribed regimen. Interestingly, the prescription of initial regimens changed over the calendar period. Over time, the prescription of regimens based in DTG and EVG increased. TDF/FTC/EVG/COBI prescription decreased but this was probably partly due to the introduction of TAF/FTC/EVG/COBI, which became available in Spain in 2016. The prescription of the other regimens (TDF/FTC/RPV, TDF/FTC+bDRV and TDF/FTC+RAL) decreased over time. These changes reflect those introduced in the Spanish HIV treatment guidelines. In 2014, all the regimens included in this study were considered preferred treatments [12]. However, since 2015 the guidelines became much more restrictive and have only recommended regimens based on integrase inhibitors as preferred, and have classified all the other regimens analyzed as alternative [1,13,14]. Despite being a preferred regimen, TDF/FTC+RAL was prescribed much less than the regimens based in DTG or EVG. This is probably due to the availability of these latter drugs as part of single tablet regimens.

Different regimens were preferentially prescribed to certain groups. The use of integrase inhibitors was more frequent among MSM. This could be in part due to patients’ preference, as MSM frequently have more access to information about HIV [8] and could be more informed about new treatments (and request them to their physicians) than other risk groups.

TDF/FTC+RAL, TDF/FTC+bDRV and TDF/FTC+DTG were more likely to be prescribed to patients with low CD4 counts. The use of TDF/FTC is probably due to the need to initiate treatment promptly when results from HLA B5701 are not yet available, and RAL and DTG could probably be used to minimize interactions in patients that might be being treated for other opportunistic diseases. Also, prevalence of resistance to integrase inhibitors is low in Spain [15,16] and these agents are frequently prescribed when resistance tests are still pending. The prescription of TDF/FTC+bDRV for patients with low CD4 counts reached borderline statistical significance, probably due to the high genetic barrier of protease inhibitors that allows their use in severely immunosuppressed patients while waiting for resistance test results, as described in earlier studies[5].

The choice of regimens varied significantly by hospital location. This is not explained by local variations in clinical characteristics of the patients, as these differences were still large after adjusting for all other factors. Possible explanations include regional variations on the physicians’ perceptions of efficacy and safety of different regimens[17], different experience on HIV treatment among the treating physicians, and institutional constraints. Although we could not assess limitations to ART prescription in all the participating hospitals for the study period, we have recently described the limitations for the prescription of antiretrovirals in the CoRIS cohort for the period 2010–2015: 54.1% of the centres had a cost limitation for the prescription of ART and 29.7% had restricted access to at least one antiretroviral or single-tablet regimen (mainly some of the newest integrase inhibitors)[18,19]. Although we could not analyze the influence of institutional constraints in the results of this study, it is possible that they could have an influence in the choice of the initial ART: our previous study demonstrated that hospitals with restricted access to at least one antiretroviral were more likely to prescribe ART regimens that were not recommended by the Spanish clinical guidelines[19].

Our study is limited by the lack of information on individual physician’s characteristics, and some clinical variables that could influence the choice of ART such as pregnancy, resistance testing or other drugs that could interact with ART. However, our strengths include the analysis of a reasonably large number of patients from a multicenter well established cohort, and the recent time period which allow us to analyze the newer treatment regimens. Our results suggest that initial ART prescriptions are influenced by clinical and demographic patient variables, and also by the prescribing hospital location.

In conclusion, in this multicenter Spanish cohort the prescription of initial ART regimens varied not only with patients’ clinical and demographic characteristics, but also with other variables such as the period of ART initiation and hospital location. Over time, regimens based on integrase inhibitors have become the most frequent choice as initial ART.
